# Automatic MRI-Based segmentation of ablation zones in spine and liver: A comparative study of network performance and influencing factors

**DOI:** 10.1007/s11548-026-03775-0

**Published:** 2026-08-19

**Authors:** Janine Rothert, Sylvia Saalfeld, Marcel Gutberlet, Frank Wacker, Daniel Düx, Bennet Hensen, Mathias Becker, Georg Rose, Georg Hille

**Affiliations:** 1 Institute for Medical Engineering, Otto-von-Guericke-University Magdeburg, Magdeburg, Germany; 2 Research Campus Stimulate, Magdeburg, Germany; 3 Institute for Medical Informatics and Artificial Intelligence, University Hospital Schleswig-Holstein Campus Kiel, Kiel, Germany; 4 Institute for Diagnostic and Interventional Radiology, Hanover Medical School, Hanover, Germany; 5 Radiology and Nuclear Medicine, Medical Faculty, University Hospital Magdeburg, Magdeburg, Germany

**Keywords:** Liver, Spinal Metastases, Thermal Ablation, Ablation Zone, Segmentation, Deep Learning

## Abstract

****Purpose**:**

Ablation therapies are a treatment option for cancer patients, particularly for conditions such as spinal metastases and liver tumors. Precisely delineating ablation zones is essential for accurately assessing treatment success. However, research in MRI-guided interventions remains limited for automated segmentation approaches and quantitative analysis.

****Methods**:**

We developed a framework for automated segmentation of ablation zones following thermal interventions. The performance was tested on two representative types of clinical cases from different clinical sites: post-ablative liver lesions and spinal metastases. Four leading neural networks (nnUNet, TransUNet, SwinUNETR, and SwinUNETR-V2) were evaluated for their segmentation accuracy in segmenting necrotic tissue. Additionally, a statistical analysis was performed to investigate the influence of an optimized image ROI selection on the segmentation performance.

****Results**:**

The nnUNet achieved the highest segmentation performance, with a Dice Similarity Coefficient of 83.3 ± 13.2% for spinal metastases and 82.2 ± 12.4% for liver lesions. The statistical analysis revealed that cropping images to a standardized ROI size provided the optimal balance between user interaction and networks’ performance.

****Conclusion**:**

The automated segmentation accuracies are comparable to the inter-rater variability observed among radiologists for spinal imaging and establish the new state-of-the-art for liver MRIs, indicating the potential to facilitate the clinical routine for interventions.

## Introduction

Cancer is responsible for one in six deaths worldwide, with an increasing incidence due to demographic changes and an aging global population. Liver cancer is the third most deadly malignancy, with over 700,000 deaths and 800,000 new cases annually [1]. Moreover, the liver is a common site for metastases for the three most prevalent cancers: lung, breast, and colorectal cancer. Cancer treatment strategies depend on tumor type and patient-specific factors. Common approaches include surgical resection, chemotherapy, and radiotherapy [2]. For patients who are ineligible for surgical resection or transplantation, ablation therapy offers a minimally invasive alternative. Techniques such as thermal ablation (e.g., microwave (MWA), radiofrequency (RFA), and cryoablation) and non-thermal ablation (e.g., irreversible electroporation) enable precise tumor destruction while preserving healthy hepatic tissue. These approaches are beneficial for patients with small (<3 cm) but centrally located tumors or those who cannot undergo more invasive procedures [2, 3].

To maximize tumor coverage and to prevent local tumor progression (LTP) [4], precise delineation of the ablation zone is essential. In clinical practice, pre-interventional imaging is often compared visually with follow-up or post-procedural images. However, a more accurate approach involves the exact segmentation of tumor and ablation zones before registering pre- and post-interventional images. Here, manual segmentation is time-consuming and introduces subjectivity, especially when estimating safety ablation margins [5]. Many studies across various anatomical regions have demonstrated that computer-assisted tools can enhance margin estimation, ultimately benefiting patient outcomes [6–8] Although magnetic resonance imaging (MRI) requires compatible materials and provides a limited interventional workspace, it offers superior soft tissue contrast compared with computed tomography (CT) while avoiding patient exposure to ionizing radiation. Consequently, our work focuses on ablation segmentation in MRI

To further complement ablation zone segmentation and investigate the methods boundaries, we extended our analysis to an additional anatomical structure: the spine and spinal metastases. The clinical incidence of spinal metastases among three commonly diagnosed cancers (breast, lung, and prostate) ranges from 10.94% in breast cancer patients to 20.04% in prostate cancer patients [9, 10]. This translates to approximately 920,000 cases of spinal metastases worldwide while osseous metastases represent the third most common metastatic site for solid tumors, with the majority occurring in the spine, particularly within the vertebrae [11].

### Related work

Computer-assisted tools have demonstrated their potential in aiding physicians during ablation therapies for both spinal [8, 12] and hepatic applications [6, 7]. While Odisio et al. [6] and Whyne et al. [12] primarily investigated CT-based imaging, Hocquelet et al. [7] and Hille et al. [8] explored MRI-based approaches. However, these studies mainly focused on the registration of pre- and post-ablation imaging, without providing segmentation accuracy metrics.

The present study focuses on the segmentation of spinal and hepatic ablation zones in MRI following thermal ablation. Despite its clinical relevance, the automated segmentation of ablation zones in MRI remains largely underexplored. To the best of the authors’ knowledge, only a limited number of studies have addressed the automatic segmentation of spinal ablation zones in vertebral metastases, most notably the work by Steffen et al. [13] and subsequent analyses based on the expanded dataset by Hürtgen et al. [14]. Steffen et al. employed both 2D and 3D UNet architectures on various MRI sequence combinations, achieving a Dice Similarity Coefficient (DSC) of 77.2 ± 15.6% (contrast-enhanced (ce)$\text {T}_1$ and $\text {T}_2$), a True Positive Rate (TPR) of 81.6 ± 15.4% (ce$\text {T}_1$ and $\text {T}_2$), and a True Negative Rate (TNR) of 99.6 ± 0.9% (ce$\text {T}_1$ only). Hille et al. [15] also reported an inter-rater variability (IRV) of 82.4 ± 5.9% DSC. For liver ablation zone segmentation, more studies have been carried out but only Hocquelet et al. [7] have investigated ablation zone segmentation in MRI. Their study employed a semi-automated active contour method directly applied by radiologists; however, it lacks a comparison against ground truth expert segmentation masks.

Most studies have predominantly relied on CT imaging and conventional segmentation techniques. For instance, Egger et al. [16] developed a semi-automated method for segmenting ablation zones in CT images following RFA of hepatocellular carcinomas, both with and without visible ablation needles. They reported a DSC of 77.0 ± 4.7% and an IRV for DSC of 88.8 ± 3.3%. Additionally, Passera et al. [17] and Wu et al. [18] employed clustering-based techniques on contrast-enhanced CT images, achieving a TPR > 90% and a TNR > 96% (Passera et al. [17]) in eight out of ten cases, and a DSC of 76 ± 3% compared to manual segmentation (Wu et al. [18]).

In summary, automated segmentation of ablation zones in MRI remains an underexplored area, particularly for liver ablation zones. Existing research is limited, with most studies focusing on CT-based methods rather than utilizing MRI’s superior soft-tissue contrast and lesion delineation capabilities. To advance this field, our study investigated state-of-the-art segmentation techniques across distinct anatomical contexts (spinal metastases and hepatic tumors) demonstrating their potential for a more generalized workflow applicable to other organ sites. Furthermore, we compared predefined patch-based approaches to holistic whole-volume segmentation to evaluate their performance differences.

## Methods

Building on these insights, the following section outlines the training procedures for the MRI-based automatic segmentation underlying our study.

### Data acquisition and preparation

**Fig. 1:**
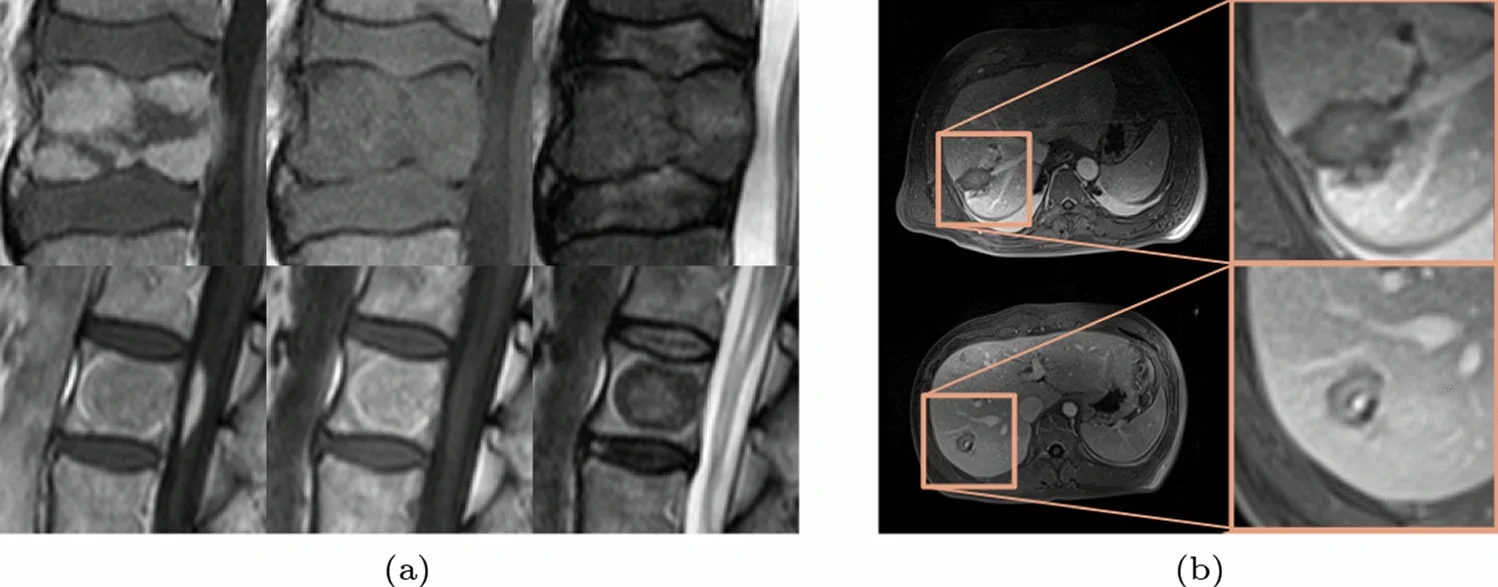
Exemplary ablation zone crops for both datasets. (a) Spinal dataset across ce$\text {T}_1$, $\text {T}_1$, and $\text {T}_2$ sequences. (b) Liver dataset in ce$\text {T}_1$, illustrating diverse appearances such as unclear boundaries, brightness variations, fluid accumulation, and ablation needles

Our method is developed on two distinct ablation scenarios, i.e., spinal and hepatic lesions. A detailed comparison is provided in Tab. 1 and exemplary ablation zone images in Fig. 1a and 1b . Reference labels were manually delineated on a slice-by-slice basis by clinical experts and subsequently reviewed under the supervision of an experienced radiologist.

**Table 1:** Comparison of the clinical aspects of spine and liver datasets

	Spine	Liver
Cancer site	Metastatic lesions within spinal vertebrae	Mostly primary tumors within the liver (11 HCC, 1 CCC, 6 metastases)
Imaging	Follow-up MRIs a few days after ablation with different contrasts (T$_1$, contrast-enhanced T$_1$, T$_2$)	Post-interventional MRI with contrast-enhanced T$_1$ sequences, partly with needles still inserted
Clinical institution	Department of Neuroradiology, University Hospital of Magdeburg	Institute for Diagnostic and Interventional Radiology, Hanover Medical School
Treatment	RFA of 35 cases from 28 patients	MWA of 28 cases from 21 patients
Clinical outcome	Mostly palliative therapy	Mostly curative therapy

The 35 spinal datasets were standardized to 64 sagittal slices using cubic interpolation for MR images and shape-preserving interpolation for segmentation masks, resulting in an in-plane resolution ranging from 0.47 mm to 1.02 mm, and inter-slice spacing between 0.77 mm and 1.29 mm. A lesion-centered bounding box (128×128×64 voxels) was defined to simulate manual localization, with nine additional randomly scaled boxes per case to increase variability (up to 256×256×64 voxels). All volumes were resampled to 128×128×64 voxels, yielding 350 samples, each with three MRI sequences as input channels. 5-fold cross-validation used 280 samples for training (28 patients), while 7 distinct patients were reserved exclusively for validation. Validation was performed only on the original image resolution.

The liver dataset consists of 28 cases with a resolution of 1.41 mm to 1.64 mm in plane and 2 mm between planes. Preprocessing for training and evaluation also includes standardized cropping to 64×64×64 voxels based on the largest segmentation mask and on maximal ablation zone size with current microwave devices [19]. Additional resizing as for the spinal data was not required, as the ablation zones were comparable in size. Here, 5-fold cross-validation resulted in a split of 22 or 23 samples for training and 5 or 6 samples for validation, depending on the fold.

### Network training

Four recent networks were evaluated for ablation zone segmentation, including CNN- and transformer-based models for 2D and 3D imaging. The 3D nnUNet [20] served as the baseline, alongside three transformer-based UNet variants for 2D [21] and 3D [22, 23] imaging, providing a comprehensive comparison across architectures. Unless otherwise specified, implementations followed the original designs and training was performed on a Linux system with an Intel Xeon CPU and NVIDIA RTX 2080 Ti GPU (12 GB) using 5-fold patient-level stratified cross-validation. The results were evaluated using the area-based metrics Dice Similarity Coefficient (DSC) and Intersection over Union (IoU), True Positive Rate (TPR) and True Negative Rate (TNR) to assess over-/under-segmentation, and the 95th percentile Hausdorff Distance (HD95) as a robust distance-based measure.

**nnUNet** The nnUNet [20] has classical UNet architectures, specifically designed for a variety of medical segmentation tasks, and consistently achieves high performance in recent segmentation challenges. It provides a robust baseline for comparing different architectures. In our study, preliminary tests suggested the 3D full-resolution configuration of nnUNet for all experiments. Each network was trained for 100 epochs with images normalized to a range of 0 to 1.

**TransUNet** The backbone of TransUNet [21] also features a UNet structure, enhanced with a vision transformer integrated at the end of the encoder. To address the limitations of our smaller dataset, we implemented additional augmentation techniques and trained each fold for 100 epochs. A major distinction compared to the other networks is its use of 2D input slices. Therefore, each input volume is divided into 64 sagittal images after the normalization of the volume grayscale values to a range between 0 and 1. The chosen default batch size is 24 slices.

**SwinUNETR & SwinUNETR-V2** SwinUNETR [22] takes a different approach from TransUNet by embedding vision transformers directly into the encoder in combination with a shifted window self-attention mechanism (swin-block). SwinUNETR-V2 [23] extends the original architecture by incorporating residual convolutional layers before and after each swin-block. To address GPU memory limitations, the batch size was set to two, and the patch size used in the shifted window mechanism for both architectures was reduced to 96×96×64 voxels. We employed a composite loss function combining DSC and Cross Entropy in accordance with the preceding approaches. Due to the increased architectural complexity and the fully 3D processing, training was extended to 200 epochs, incorporating 3D augmentation strategies. Channel-wise normalization was applied using the mean and standard deviation values from the original implementation.

## Results

**Fig. 2:**
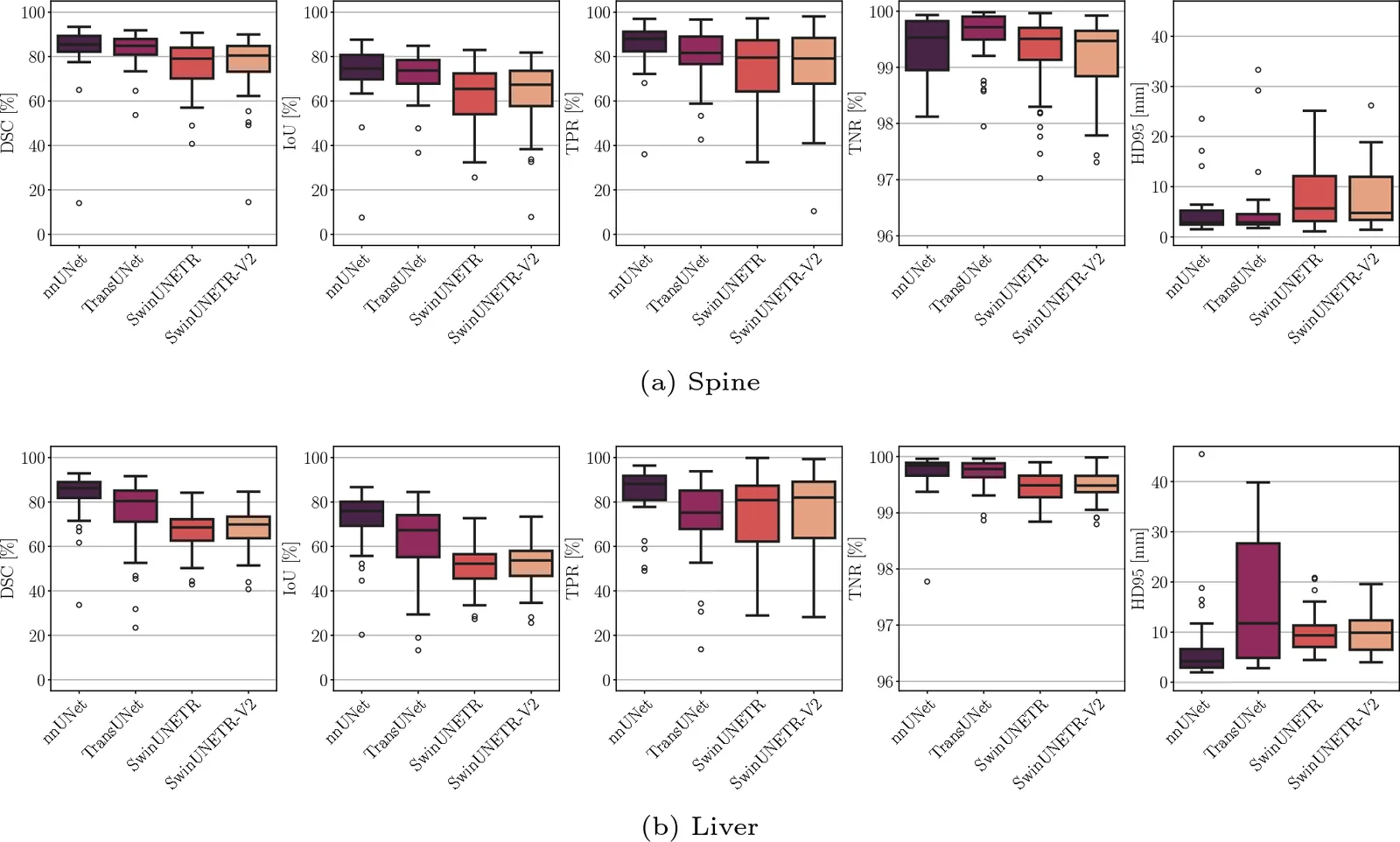
Boxplots show five performance metrics (DSC, IoU, TPR, TNR, HD95) across four networks: nnUNet, TransUNet, SwinUNETR, and SwinUNETR-V2

Table 2 and the corresponding boxplots (Fig. 2a, 2b ) summarize the average performance of four networks on both datasets. nnUNet achieved the best results, with DSCs of 83.3 ± 13.2% for spine and 82.2 ± 12.4% for liver data. TransUNet performed comparably on spinal images, while all transformer-based models showed reduced accuracy, particularly for liver segmentation, likely due to limited preprocessing and dataset size. Overall, none outperformed nnUNet.

**Table 2:** The table summarizes cross-validation results for different network architectures. Spinal ablation segmentation used the optimal ceT$_1$, T$_1$ and T$_2$ combination [14], while liver cases only used ceT$_1$

	DSC [%] ↑	IoU [%] ↑	TPR [%] ↑	TNR [%] ↑	HD95[mm] ↓
Spine					
nnUNet	**83.3 ± 13.2**	**72.8 ± 13.7**	**85.1 ± 11.2**	99.4 ± 0.6	**4.63 ± 4.61**
TransUNet	83.2 ± 7.9	71.9 ± 10.5	80.0 ± 12.9	**99.6 ± 0.5**	5.24 ± 6.84
SwinUNTER	76.1 ± 11.6	62.6 ± 14.0	75.6 ± 15.8	99.2 ± 0.8	7.73 ± 6.00
SwinUNETR-V2	75.8 ± 14.7	62.9 ± 15.6	76.5 ± 17.3	99.2 ± 0.7	7.58 ± 6.33
Liver					
nnUNet	**82.2 ± 12.4**	**71.3 ± 14.8**	**83.7 ± 13.1**	99.7 ± 0.4	**7.35 ± 8.75**
TransUNet	73.7 ± 17.8	61.0 ± 19.3	71.8 ± 19.2	**99.7 ± 0.3**	15.69 ± 12.24
SwinUNETR	66.4 ± 10.5	50.6 ± 11.3	73.7 ± 19.0	99.5 ± 0.3	10.14 ± 4.41
SwinUNETR-V2	68.0 ± 10.8	52.4 ± 11.9	75.4 ± 18.1	99.5 ± 0.3	10.06 ± 4.24

**Fig. 3:**
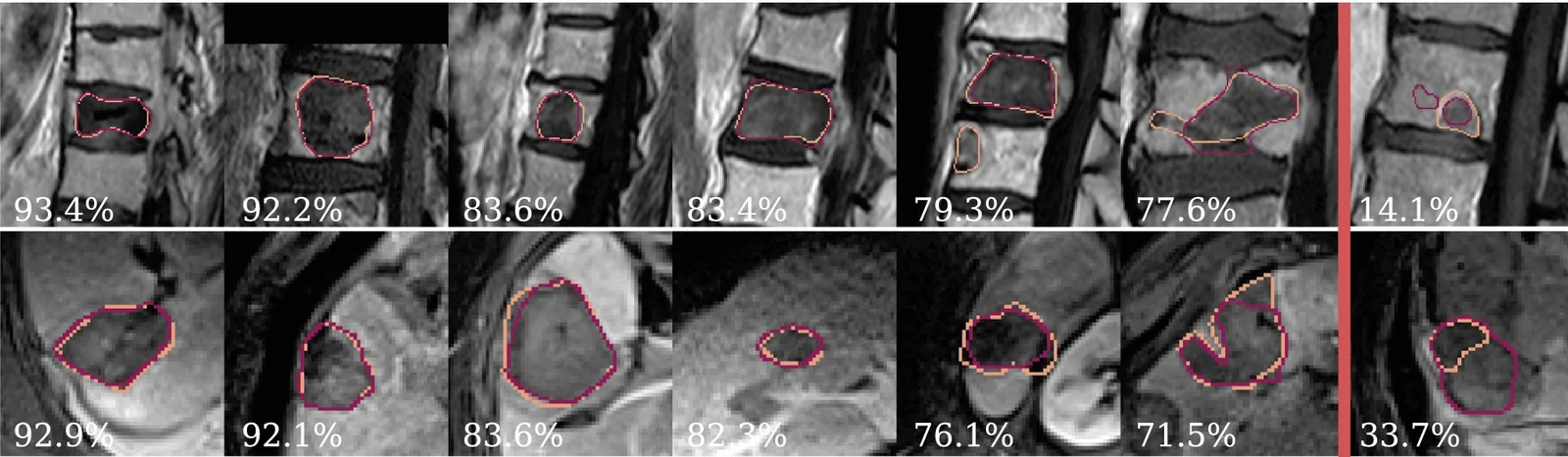
Segmentation results on $\text {ceT}_1$ are shown for representative spinal (upper) and liver (lower) cases with high, average, and below IRV DSC. Predictions (dark purple) are compared with ground truth (light orange), and each case’s DSC is shown in the lower-left corner. The lowest-performing outlier is shown right, separated by a red line

Representative spine and liver cases with best, average, and below IRV performance by the nnUNet are shown in Fig. 3, illustrating generally good alignment. The outlier cases highlight challenges such as unconventional ablation shapes or limited visual separation from adjacent prior ablations or lesions.

**Influence of ROI size on segmentation performance**

To validate the patch-based approach for a clinical segmentation pipeline, we evaluated three different methodologies using the best-performing architecture and baseline model, namely nnUNet, while training with the same cross-validation approach outlined in Sec. Network training. Each experiment incorporating decreasing ROI sizes while requiring additional cropping information. These approaches included: (1) segmentation of whole image volumes, where multiple ablation zones were merged into a single mask (semantic segmentation), (2) volume crops as described in Sec. Data acquisition and preparation, and (3) patches defined by the bounding box of the ablation zone, with an additional 25% margin in each dimension. The results of this statistical analysis are presented in Tab. 3.

**Table 3:** The table summarizes cross-validation results for different ROI sizes and the effect of detailed user input on segmentation accuracy, using the best-performing network (nnUNet) from Sec. Results

	DSC [%] ↑	IoU [%] ↑	TPR [%] ↑	TNR [%] ↑	HD95 [mm] ↓
Spine					
Whole volume	73.5 ± 21.4	61.5 ± 21.0	73.4 ± 23.8	**99.9 ± 0.0**	34.02 ± 61.09
Volume crops (128 × 128)	83.3 ± 13.2	72.8 ± 13.7	85.1 ± 11.2	99.4 ± 0.6	4.63 ± 4.61
Bounding Box (+25%)	**84.8 ± 8.6**	**74.4 ± 11.0**	**87.4 ± 8.5**	97.2 ± 2.0	**4.07 ± 3.68**
Liver					
Whole volume	66.1 ± 27.7	54.7 ± 27.3	63.5 ± 28.7	∼ **1.0 ± 0.0**	29.65 ± 36.60
Volume crops (64 × 64)	82.2 ± 12.4	71.3 ± 14.8	83.7 ± 13.1	99.7 ± 0.4	7.35 ± 8.75
Bounding Box (+25%)	**86.3 ± 7.4**	**76.5 ± 9.6**	**88.0 ± 4.1**	98.1 ± 2.1	**5.21 ± 4.57**

While the whole-volume approach enabled fully automated segmentation without the need for additional user input, it was unable to achieve satisfactory accuracy, particularly for liver ablation zones reflected by significant different results to the patch-based approaches (p-value to cropped volumes for independent two-sample t-test: 0.0277 (spine), 0.0089 (liver) and bounding box 0.0055 (spine), 0.006 (liver)). This limitation likely arises from the presence of additional metastases or previously treated lesions, which introduced ambiguity in network learning and increased the standard deviation of the results besides the increased search space with an imbalanced ratio between relevant and irrelevant image information. As expected, the smallest patch from the expanded bounding box yielded the highest segmentation accuracy. However, this approach required defining the lesion extend in three dimensions, increasing both time and effort compared to the slightly inferior yet more practical standardized patch-based method with no significant difference (p-values for independent two-sample t-test: 0.5626 (spine), 0.1404 (liver)). Consequently, the latter demonstrated the best trade-off between segmentation accuracy and the need for additional localization information, which could be derived automatically from the needle position, the original tumor site, or a single user interaction.

Building on these findings, we suggest a workflow for the assessment of ablation zones via accurate segmentation, based on a clinical setting where interventionalists treat one lesion at a time. First, ROI selection identifies the ablation zone as a region of interest (ROI), chosen either manually by a physician or automatically from prior tumor or needle position information, and then cropped to a standardized patch size for improved segmentation. Second, segmentation assigns each anatomical region a dedicated model that processes cropped MR images to generate segmentation masks, forming the core of accurate and reliable ablation zone delineation. Finally, analysis uses the segmentation mask for treatment outcome evaluation or comparison with pre-interventional imaging, with further exploration left for future work.

## Discussion

In this study, we evaluated the segmentation performance of four neural networks for delineating ablation regions following thermal ablation therapies. The liver and spine datasets cover a variety of clinical and technical aspects, making them suitable benchmarks for assessing the current capabilities of deep learning models in medical image segmentation. Across both datasets, state-of-the-art DSC were achieved with nnUNet: 83.3 ± 13.2% for the spine dataset, surpassing the performance of Steffen et al. [13] with a DSC of 77.2 ± 15.6% and 82.2 ± 12.4% for the liver dataset, where no comparable MRI-based segmentation accuracy has been reported, establishing our result as a baseline. In contrast, previous studies investigating liver ablation zone delineation from smaller CT imaging datasets have reported lower segmentation accuracies, i.e., 77 ± 4.7% DSC across twelve clinical cases [16], and 76 ± 3% DSC in a preclinical swine model without tumors [18]. Although these results show lower variability, the absence of tumors in [18] simplifies the delineation task, as there is no need to differentiate between residual tumor tissue and ablation areas. Passera et al. [17] reported a higher true positive rate (TPR) of 90%, but a lower true negative rate (TNR) of 96%, indicating increased oversegmentation. In comparison, our results exhibit a more balanced performance with a TPR of 83.7% and a TNR of 99.7%, suggesting minimal oversegmentation and greater precision in necrotic tissue delineation.

Notably, the performance on our spine dataset is comparable to the IRV of 82.4 ± 5.9%, as reported in [15], which can be considered an estimate of the achievable performance ceiling for learning-based segmentation approaches. For liver MRI, no direct IRV values for ablation zone segmentation are available in the literature. However, a reference DSC of 88.8 ± 3.3% has been reported in CT imaging [16]. This value currently surpasses the performance of the proposed model, which may be attributed to several factors: the presence of outliers, the inclusion of untreated lesions that introduced ambiguity during both training and inference, and the limited availability of complementary imaging information due to the use of a single MRI sequence (ce$\text {T}_1$). Incorporating additional sequences in future studies could mitigate these limitations. However, the ce$\text {T}_1$ MRI sequence plays a critical role in the segmentation of ablation zones and lesions, particularly in spinal and brain imaging [14, 24] most likely also transferring to liver imaging as well.

Notably, the HD95 values observed in both scenarios (4.63 mm for the spine and 7.35 mm for the liver) are of a similar magnitude to the 5 mm safety margin applied around the tumor. Consequently, larger localized boundary deviations may have a relevant impact on the resulting margin. However, because HD95 quantifies the 95th percentile of surface distance errors rather than the overall average segmentation accuracy, this metric is particularly sensitive to more pronounced local discrepancies, even when the overall segmentation quality remains high, as indicated by the high DSC values.

Limitations may arise from the use of a standardized patches, particularly in cases where multiple adjacent lesions are present within the same image. This can complicate the model’s ability to distinguish between distinct pathological zones. Therefore, the network was specifically trained to segment only the actively treated and most centrally located ablation zone. This strategy reflects clinical practice, which prioritizes evaluating the currently treated lesion rather than all lesions simultaneously. Another limitation is the use of separate networks for segmenting spinal and liver ablation zones, rather than employing a unified model. Although a combined network may appear more generalizable, its performance would likely decline because the spine and liver differ in anatomical context and tissue characteristics. Moreover, extending a unified model to cover additional ablation sites would require complete retraining. In contrast, separate networks allow for greater modularity and flexibility, enabling the integration of new anatomical regions into the tool without affecting existing models especially in cases like ours, where the dataset size is limited and each additional data point could increase the robustness.

In contrast to the organ-specific models, we didn’t separate models segmenting ablation with or without inserted needles as represented by the liver dataset. Additional analysis showed, that cases with a visible needle (n=17) achieved a higher average DSC (84.78 ± 6.90 %) compared to cases without a needle (n=11) (78.18 ± 17.64 %). However, this comparison should be interpreted with caution, as the no-needle group predominantly consists of more complex cases with multiple tumors per patient. In these patients, only the currently treated lesion contains a needle, while other lesions do not, resulting in an uneven distribution of case complexity between groups. Although the results may suggest that the needle provides additional spatial information that helps the network identify the ablation location more accurately, training separate networks for needle and no-needle cases would not be practical due to the clinical workflow of sequentially treating multiple tumors and evaluating treatment success only at the end with an MRI. Future work employing explainable AI approaches and advanced visualization techniques could provide further insights into how the network utilizes needle-related information.

A potential medical limitation is associated with ablation induced tissue shrinkage, which may affect both manual and automated delineations by reducing the apparent pathological volume of the ablated region, resulting in a systematic underestimation of the true ablation extent relative to pre-operative imaging. However, this bias may be less critical in clinical decision-making, as it is generally more relevant to ensure complete ablation coverage than to risk overestimation of the treated area.

Future work will focus on developing a tool for integration into clinical routine. Here, a critical step is the incorporation of both pre-interventional and post-interventional imaging, including image registration and initial tumor segmentation. This enables direct assessment of tumor coverage by the ablation zone, as well as 3D distance visualization to ensure a consistent 5 mm safety margin in all directions. Compared to conventional 2D slice-by-slice evaluation, this method offers greater spatial accuracy while the final decision making and manual segmentation inspection will be subject to human experts. An additional advantage lies in its potential intraoperative application: real-time assessment using a needle-based reference could help identify residual tumor tissue, allowing immediate re-ablation and potentially avoiding the need for a second intervention and LTP, particularly important in liver ablation for curative intent.

## Conclusion

In summary, we demonstrated state-of-the-art performance across a representative combination of datasets from different clinical sites for ablation zone segmentation after a thermal ablation intervention in MRI. The proposed segmentation pipeline, incorporating a ROI selection strategy, proved effective for both microwave and radiofrequency ablation cases, and showed robust performance in anatomically diverse tissues, including homogeneous liver parenchyma and solid spinal bone structures. Future work toward the goal of an applicable tool can create an improved ablation zone assessment and therefore an optimized clinical workflow.
